# Graft-Versus-Host Disease Prophylaxis after Transplantation: A Network Meta-Analysis

**DOI:** 10.1371/journal.pone.0114735

**Published:** 2014-12-08

**Authors:** Panayiotis D. Ziakas, Fainareti N. Zervou, Ioannis M. Zacharioudakis, Eleftherios Mylonakis

**Affiliations:** Division of Infectious Diseases, Warren Alpert Medical School of Brown University, Providence, Rhode Island, United States of America; UNIFESP Federal University of São Paulo, Brazil

## Abstract

**Background:**

Graft-versus-host Disease (GvHD) prophylaxis after allogeneic hematopoietic stem-cell transplantation (HSCT) is an ongoing effort but relative effects of different policies are not systematically explored.

**Methods:**

We systematically reviewed 30-year evidence on GvHD prophylaxis and quantified the relative effect of different policies using a network meta-analysis. We searched PubMed and the Cochrane Library for randomized studies on the topic. The primary outcome of interest was grade II-IV acute GvHD over 0 or I (with odds ratio OR <1 denoting benefit).

**Findings:**

Thirty-three eligible studies that enrolled 3,440 patients (published up to June 2014), provided data on seven immunosuppressive drugs namely cyclosporin A (CsA), methotrexate (MTX), anti-thymocyte globulin (ATG), mycophenolate mofetil (MMF), tacrolimus, sirolimus or corticosteroids and their combinations to calculate 14 direct and 21 indirect effects. The majority of trials (32/33) referred to myeloablative conditioning and sibling transplants (25/33). Tacrolimus/MTX (OR 0.44; 95% 0.27–0.70, number needed to treat to benefit, i.e. to avert a case of II-IV GvHD, NNTB = 5) and ATG/CsA/MTX (OR 0.45; 95%CI 0.26–0.78; NNTB = 5) were superior over CsA/MTX. ATG/CsA/MTX did not differ from tacrolimus/MTX (indirect evidence). Sirolimus-based prophylaxis outperformed CsA/MTX (OR 0.10; 95%CI 0.02–0.49, NNTB = 4) and marginally outperformed tacrolimus/MTX (OR 0.22; 95%CI 0.05–1.11). Add-on corticosteroids had no benefit over CsA/MTX.

**Conclusions:**

Tacrolimus/MTX and ATG/CsA/MTX were the outperformers over CsA/MTX, but sirolimus-based regimens showed also potential. More randomized data are needed for reduced-intensity conditioning, as well as for MMF and sirolimus-containing regimens.

## Introduction

The progress in the field of hematopoietic stem transplantation (HSCT) has resulted in a substantial rise in eligible patients and expanded therapeutic indications of HSCT. In 2010 only, over 12,000 patients received allogeneic transplant across Europe and approximately 7,000 in the US, figures reported by the European Group of Blood and Marrow Transplantation (EBMT) [1] and the Center for International Blood and Marrow Transplant Research (CIBMTR) [2], respectively. Despite the documented progress, graft versus host disease (GvHD) still remains an important constraint in allogeneic HSCT that partially hampers ongoing efforts to expand the pool of eligible candidates. Acute GvHD correlates inversely with both overall survival and treatment related mortality, and II-IV grade represents a clear cut-off in prognosis [3], [4].

Morbidity remains high, treatment is difficult and prevention strategies are far away from being considered optimal [5]. It was not until recently that the European Group for Blood and Marrow Transplantation and the European LeukemiaNet working group (EBMT-ELN) have published pertinent recommendations for GvHD, aiming to standardize prevention and treatment policies [5]. Optimization of prevention for GvHD remains an ongoing effort, as retrospective data analysis – even for data derived from randomized studies – suffers from substantial clinical heterogeneity between studies and inconsistencies of assigned pharmacologic interventions. In that context, we systematically reviewed pertinent randomized data, in order to summarize the relative effects of assigned protocols on GvHD prophylaxis using a network meta-analysis of direct and indirect comparisons.

## Methods

We searched PubMed and The Cochrane Library databases for pertinent randomized trials. Last access was on June 13, 2014. The search terms were: “(GvHD OR graft versus host) AND (randomized OR randomised)”. We further scrutinized bibliography of eligible articles for additional studies on the topic. We complemented our search to include the American Society of Hematology (2004-2013) and the European Hematology Association (2006-2014) proceedings for additional randomized trials on the topic. Language restriction was not imposed. We followed the PRISMA guidelines (S1 Checklist in S1 Appendix).

A randomized trial on HSCT was deemed eligible provided that it met all the following conditions: (1) it randomized prophylactic schemes for GvHD, (2) reported acute GvHD as an outcome of interest, and, (3) randomized immunosuppressive drugs or drug combinations that are included in the recent EBMT-ELN working group consensus for a standardized practice in HSCT [5]. A trial was excluded from analysis if it had no extractable data on acute GvHD after prophylaxis, compared different dosing or formulations of the same pharmacologic agent, or used *post hoc* or historical arms for comparison. In case of follow-up, extension or overlapping studies, only the first published article was included. Studies outside the prophylactic setting, such as upfront or salvage therapies for acute GvHD were not considered.

Three reviewers (PDZ, IMZ and FNZ) screened titles and abstracts for relevance to the topic. All potentially relevant publications were independently evaluated in full text by the same authors. The following information was sought: first author, publication year, country of origin, sample size, median age, underlying condition, donor type, setting (myeloablative or reduced-intensity conditioning, RIC), conditioning regimens, total body irradiation, GvHD prophylaxis stratification and risk of acute GvHD. The primary outcome of interest was acute GvHD to day +100, dichotomized as II-IV grade over 0-I grading. We chose II-IV over 0-I because this stratification represents a clear-cut off in prognosis [3], [4] and the clinical cut-off to initiate GvHD treatment [5]. For completeness of the analysis we added III-IV as a secondary outcome.

The quality of individual studies was graded using the Cochrane Collaboration's tool for assessing the risk of bias, with the use of 5 pertinent items: random sequence generation and allocation concealment (for selection bias), blinding of participants and personnel (for performance bias), incomplete outcome (attrition bias), and selective reporting (for selection bias) [6].

The evidence synthesis is a network of comparisons, consisting of (a) pairwise direct effects of the prophylactic regimens, and, (b) indirect effects between two treatments against a common comparator [7]. For direct comparisons, the random-effects pooled Odds Ratio (OR), according to DerSimonian and Laird, was calculated and 95% precision estimates were reported [8]. We quantified statistical heterogeneity using the Cochran's Q test and I^2^ metric [9]. Evidence for small study effects was sought using the Harbord-Egger test [10].

We applied the Grading of Recommendations Assessment, Development and Evaluation (GRADE) classification criteria (accessible at: http://www.gradeworkinggroup.org/publications/JCE_series.htm) as previously described [11], to validate the quality of evidence regarding the risk of acute GvHD. The GRADE rating aims to explore the magnitude of confidence we have regarding our effect estimations, ranking them in descending order as high, moderate, low and very low [12]. In this context, when evidence is deemed as “high” quality, the authors are very confident that the estimates represent the true effect, as opposed to “very low” which implies that the true effect may actually differ from our estimates. The effects derived from randomized trials are initially ranked as “high” but may be downgraded in the presence of up to five limitations, namely the risk of bias, inconsistency, indirectness, imprecision and publication bias [13]. When treatments were not compared directly, effects were calculated using an indirect approach. Two competing strategies, A and B, are indirectly compared using the equation ln(OR_AB_) = ln(OR_AC_)−ln(OR_BC_), where C is an intermediate strategy with which both A and B are directly compared (the common comparator) [14]–[16]. The corresponding 95% CIs are computed assuming asymptotic normality and lack of covariance as described previously [14]–[17] Also, we reported the number needed to treat to benefit (NNTB) i.e. to avert a case of II-IV GvHD, and number needed to treat to harm (NNTH) i.e. to develop II-IV GvHD [18], [19]. Stata v13 (College Station, TX, USA) was used for data analysis and graphs of direct comparisons.

## Results

Initial search yielded 769 citations from PubMed and 694 citations from the Cochrane Library. After title and abstract screening of 1,028 non-duplicate publications, 975 were excluded on the basis of relevance and a total of 53 articles were retrieved for full-text evaluation. Twenty one were not deemed eligible because they presented extension/overlapping data (n = 12), randomized drugs not advocated by the EBMT-ELN working group (n = 4) [5], or evaluated different dose/schedule of the same drug (n = 4). Also, one study was a matched-pair analysis (not truly randomized). No additional studies were found from conference proceedings.

Thirty two articles [20]–[51] remained eligible for final analysis on acute GvHD prevention, providing data 3,440 patients from 33 randomized studies (S1 Flow-Chart in S1 Appendix). The majority of studies (25/33, 76%) referred to sibling donors and myeloablative setting (32/33, 97%). Four studies (12%) presented data on both unrelated and sibling donors, and 4 studies (12%) on unrelated donors solely. Of note is that one study had 40% of patients receiving RIC and no study had randomized solely RIC transplants [25]. Bone marrow transplants were used in 26 studies, peripheral blood stem-cells in 2, and 5 enrolled patients receiving bone marrow or peripheral blood stem-cells.

The randomized interventions included the calcineurin inhibitors cyclosporine A (CsA) and tacrolimus, the antimetabolite methotrexate (MTX), the mTOR inhibitor sirolimus, the inosine monophosphate dehydrogenase inhibitor mycophenolate mofetil (MMF) as well as anti-thymocyte globulin (ATG) and corticosteroids. Systemic corticosteroids (Pse) referred to methylprednisolone, prednisolone and/or prednisone. Beclomethasone dipropionate (BDP) was examined separately due to the lack of systemic effects [22]. Study demographics, setting, randomized interventions and outcomes are summarized in Table 1 and the detailed study characteristics are shown on S1 Table S1 Appendix.

**Table 1:** Summary of included studies and stratified prophylaxis data on outcome.

		Arm 1				Arm 2			
Author	Year	N	Prophylaxis	GvHD II-IV	GvHD III-IV	N	Prophylaxis	GvHD II-IV	GvHD III-IV
Torres A [46]	1989	31	MTX	12	2	26	CsA	12	4
Ringdén O [44]	1986	27	MTX	6	4	30	CsA	12	6
Biggs JC [42]	1986	16	MTX	3	NR	20	CsA	9	NR
Storb R [45]	1985	23	MTX	11	NR	25	CsA	11	NR
Deeg HJ [40]	1985	39	MTX	22	17	36	CsA	12	6
Storb R [41]	1986	23	MTX	13	9	21	CsA/MTX	3	0
Hiraoka A [30]	2001	66	tacrolimus+/-MTX	12	6	65	CsA+/MTX	31	14
Nash RA [32]	2000	90	tacrolimus/MTX	46	16	90	CsA/MTX	63	23
Ratanatharathorn V [35]	1998	165	tacrolimus/MTX	53	22	164	CsA/MTX	73	28
Lee KH [28]	2004	40	CsA	8	2	40	CsA/MTX	8	5
Locatelli F [33]	2000	32	CsA	12	0	37	CsA/MTX	11	1
Zikos P [36]	1998	28	CsA	17	2	32	CsA/MTX	11	0
Mrsic M [47]	1990	39	CsA	20	NR	37	CsA/MTX	10	NR
Storb R [43]	1986	50	CsA	27	12	43	CsA/MTX	14	3
Ruutu T [31]	2000	53	MP/CsA/MTX	7	3	55	CsA/MTX	20	9
Chao NJ [34]	2000	90	pse/CsA/MTX	16	7	96	CsA/MTX	19	10
Atkinson K [49]	1991	21	pse/CsA/MTX	2	NR	20	CsA/MTX	3	NR
Storb R [48]	1990	73	pse/CsA/MTX	33	18	74	CsA/MTX	27	15
Martin PJ [22]	2012	92	BDP/tacrolimus/MTX	61	20	46	tacrolimus/MTX	31	14
Deeg HJ [37]	1997	61	MP/CsA	37	21	59	CsA	44	24
Bacigalupo A [25]	2010	84	ATG/CsA/MTX	NR	4	86	CsA/MTX	NR	13
Finke J [24]	2009	103	ATG/CsA/MTX	34	12	98	CsA/MTX	51	25
Champlin RE [26]	2007	70	ATG/CsA/MTX	8	NR	60	CsA/MTX	11	NR
Bacigalupo A [29]	2001	29	ATG/CsA/MTX	20	12	25	CsA/MTX	18	9
Bacigalupo A [29]	2001	27	ATG/CsA/MTX	10	3	28	CsA/MTX	22	14
Doney KC [50]	1981	30	ATG/MTX	8	NR	42	MTX	9	NR
Weiden PL [51]	1979	29	ATG/MTX	5	NR	27	MTX	2	NR
Pulsipher MA [20]	2014	73	sirolimus/tacrolimus/MTX	13	7	70	tacrolimus/MTX	22	9
Pidala J [21]	2012	37	sirolimus/tacrolimus	16	5	37	tacrolimus/MTX	33	4
Perkins J [23]	2010	42	MMF/tacrolimus	33	8	47	tacrolimus/MTX	37	2
Bolwell B [27]	2004	21	MMF/CsA	10	NR	19	CsA/MTX	7	NR
Chao NJ [38]	1993	75	pse/CsA/MTX	7	NR	74	pse/CsA	17	NR
Ramsey NK [39]	1982	32	pse/ATG/MTX	3	1	35	MTX	11	6

(The complete study characteristics are available on S1 Table in S1 Appendix).

CsA = cyclosporine A; MTX = methotrexate; ATG = antithymocyte globulin; MP = methyl-prednisolone; Pse = systemic corticosteroid (prednisolone or methylprednisolone); BDP =  oral beclomethasone propionate. MMF =  mycophenolate mofetil, NR = not reported.

### Quality Assessment

The quality assessment of individual studies is shown on S2 Table in S1 Appendix, stratified for the pairwise comparisons. All randomized studies were deemed of adequate quality (low or unclear risk of bias) to be included in the analysis. Across each stratum, the majority of studies had also low or unclear risk of bias, and all but one direct comparisons were considered free of serious limitation to warrant downgrading the level of evidence (S3 and S4 Tables in S1 Appendix).

### Direct effects

**Figure 1:**
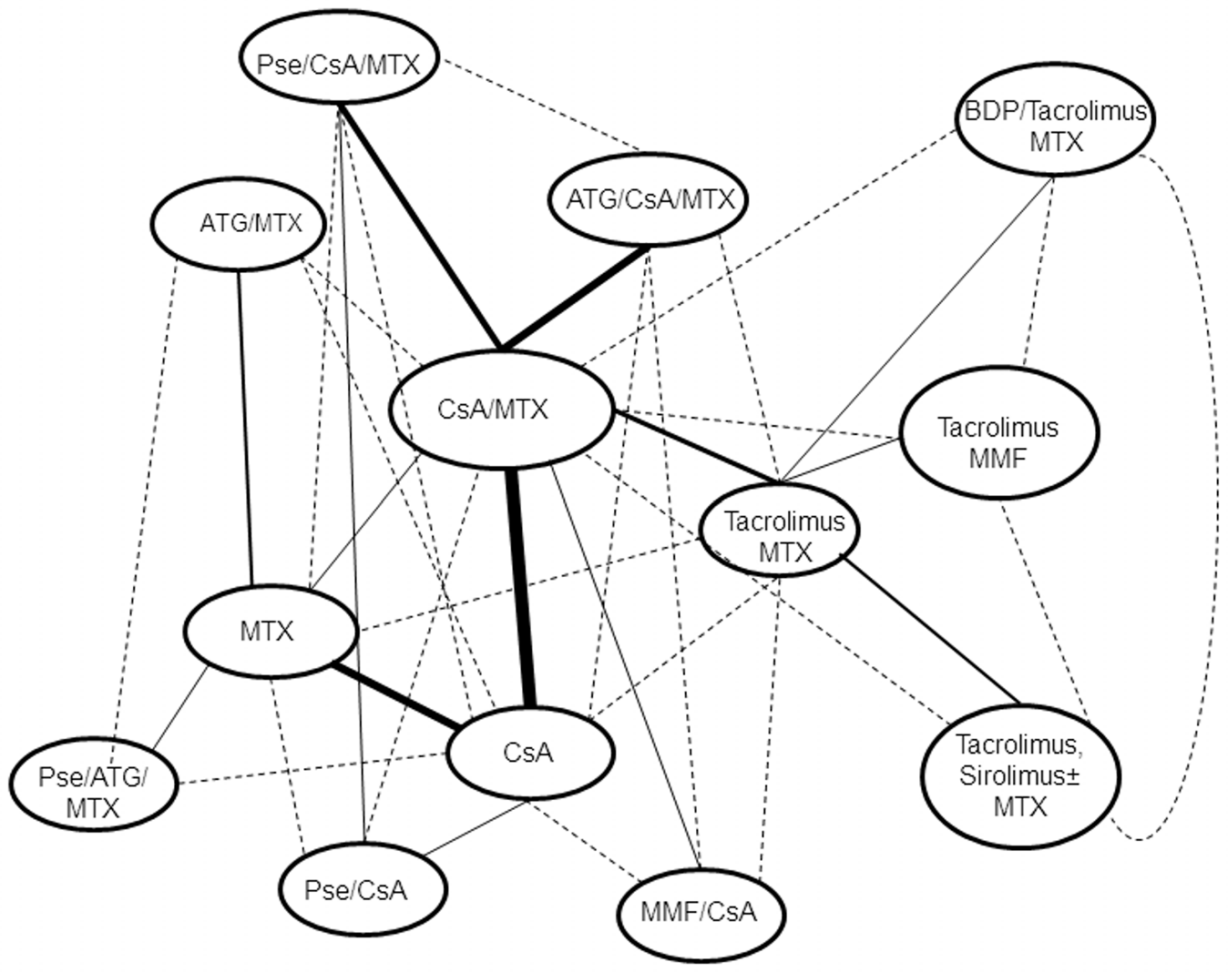
Network of direct (solid lines) and indirect comparisons (dashed lines) of different treatments evaluated for acute GvHD prophylaxis. The thickness of connecting lines is proportional to the number of available direct comparisons.

**Figure 2:**
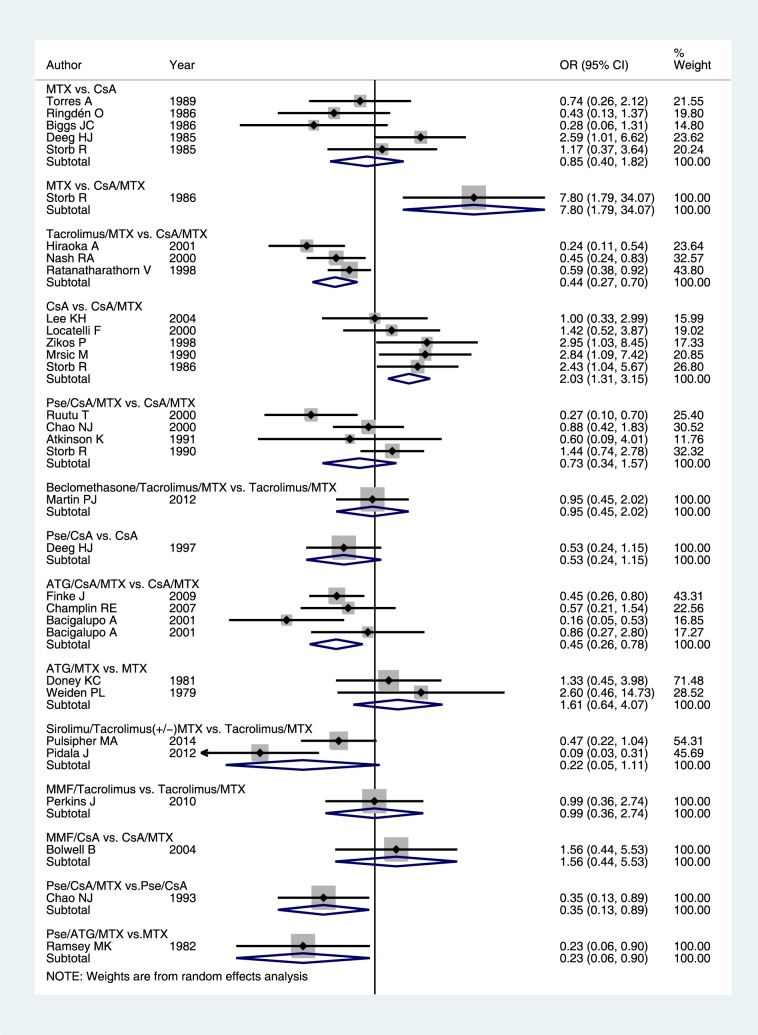
Forest plot of direct comparisons for the primary outcome (II-IV GvHD).

Direct effects were derived from 14 pairwise comparisons. The network of comparisons is shown in Fig. 1 (solid lines). The CsA/MTX combination was compared over CsA monotherapy, MTX monotherapy, tacrolimus/MTX, ATG/CsA/MTX or Pse/CsA/MTX; CsA monotherapy was compared over MMF/CsA, MP/CsA or MTX monotherapy; ATG/MTX and Pse/ATG/MTX were compared over MTX monotherapy; Pse/CsA/MTX over Pse/CsA; tacrolimus/MTX was compared over tacrolimus/sirolimus (±MTX), tacrolimus/MMF or beclomethasone (BPD)/tacrolimus/MTX. The relative effects are shown in Fig. 2, and their grading of evidence on S3 and S4 Tables in S1 Appendix.

The use of MTX over CsA monotherapy [40], [42], [44]–[46] had no significant impact on II-IV GvHD risk (OR 0.85; 95%CI 0.40–1.82; I^2^ = 55.2%), an effect derived exclusively from studies in the myeloablative setting with sibling donors and bone marrow transplants. The estimates did not alter (OR 0.76; 95%CI 0.29–2.03) after excluding the sole study exclusively on chronic myelogenous leukemia. MTX was inferior to CsA/MTX combination (OR 7.8; 95% CI 1.79–34.07, NNH = 3) in a single trial exclusively in aplastic anemia [41].

The use of tacrolimus/MTX over CsA/MTX [30], [32], [35] was associated with significant reduction of acute II-IV GvHD (OR 0.44; 95%CI 0.27–0.70, NNTB = 5; I^2^ = 45.1%), with high quality of evidence. All contributing studies used myeloablative conditioning, but differed in donor type. The association persisted (OR 0.35; 95%CI 0.19–0.63) after exclusion of [35] that showed imbalance in baseline disease characteristics (advanced or non-advanced malignancy) that potentially affected survival. The use of CSA monotherapy over CsA/MTX combination [28], [33], [36], [43], [47] significantly increased the risk of II-IV GvHD (OR 2.03; 95%CI 1.31–3.15, NNTH = 6), an effect consistent across studies (I^2^ = 0). The effect was derived from studies with comparable setting (myeloablative conditioning, bone marrow allografts and sibling donors), with the exception of one study that randomized solely aplastic anemia transplants [33].

A total of six studies examined the use of add-on corticosteroids to prophylaxis regimens, four as add-on to CsA/MTX combination [31], [34], [48], [49], one [22] as add-on to tacrolimus/MTX combination, and one as add-on to CsA [37]. Add-on corticosteroids to CsA/MTX (OR 0.73, 95%CI 0.34–1.57; I^2^ = 63%) showed no benefit regarding II-IV GvHD. The pooled effect was derived from four studies in myeloablative setting, using bone marrow transplants from sibling donors. One study enrolled exclusively adult patients [31] and the remaining three enrolled both pediatric and adult patients. After excluding [31], the effect remained insignificant (OR 1.11; 95%CI 0.69–1.78) and was consistent across studies (I^2^ = 0). Add-on corticosteroids to tacrolimus/MTX (OR 0.95;0.45–2.02) or to CsA monotherapy (OR 0.53; 95% 0.24–1.15) had no benefit regarding II-IV GvHD. It should be noted that one study [22] used oral beclomethasone propionate (BDP), which is regarded as having minimal systemic effects.

Six articles examined the use of add-on ATG to prophylaxis, four as add-on to CSA/MTX combination [24]–[26], [29] and the remaining two [50], [51] as add-on to MTX monotherapy. ATG add-on to CSA/MTX prevented acute GvHD (OR 0.45; 95%CI 0.26–0.78; NNB = 5; I^2^ = 30.1%), an association of high quality evidence (effect derived from four studies). Three out of 4 studies used rabbit ATG. The association did not alter after excluding the single study using equine ATG [26] (OR 0.41; 95%CI 0.19–0.89; I^2^ = 0%). ATG add-on to MTX monotherapy had no effect (OR 1.61; 95% CI 0.64–4.07), with the two consisting trials using equine ATG. Also, two trials on sirolimus/tacrolimus combinations for acute GvHD prophylaxis over tacrolimus/MTX [20], [21] suggested protection (OR 0.22; 95%CI 0.05–1.11, NNTB = 5, effect of marginal significance).

MMF-based combinations were not pooled to calculate direct effects [23], [27], as they were deemed highly heterogeneous with regard to donor type, donor source and assigned prophylaxis. MMF/tacrolimus was comparable to tacrolimus/MTX (OR 0.99; 95%CI 0.36–2.74) [23]; MMF/CsA was also comparable to CsA/MTX (OR 1.56;95%CI 0.44–5.53) [27]. The triple combination Pse/CsA/MTX was superior over Pse/CsA (OR 0.35; 0.13–0.89, NNTB = 7) [38] and Pse/ATG/MTX was superior to MTX monotherapy (OR 0.23; 95% CI 0.06–0.90, NNTB = 5) [39], both effects derived from single trials.

**Figure 3:**
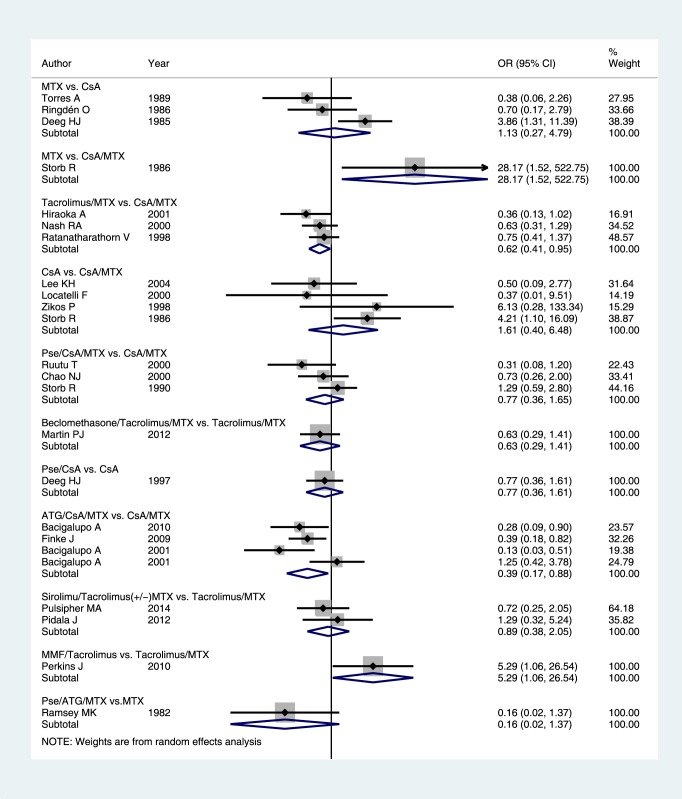
Forest plot of direct comparisons for the secondary outcome (III-IV GvHD).

Direct effects for the secondary outcome (III-IV GvHD) are presented in Fig. 3. The tacrolimus/MTX regimen was superior to CsA/MTX (OR 0.62; 95%CI 0.41–0.95; NNTB = 12), an effect consistent across studies (I^2^ = 0). ATG add-on to CsA/MTX prevented III-IV GVHD (OR 0.39; 95%CI 0.17–0.88;I^2^ = 58%), an effect derived solely from studies using rabbit ATG. In subgroup analysis, this effect was more pronounced across the two studies that used ≥15 mg/kg total dose of ATG (OR 0.26; 95%CI 0.09–0.74). MTX was inferior to CsA/MTX while MMF/tacrolimus may have resulted in increased III-IV GvHD over tacrolimus/MTX. The remaining comparisons were not statistically significant (Fig. 3).

### Indirect Effects

A total of 21 indirect comparisons were available for the primary outcome. The network of indirect comparisons is shown in Fig. 1 (dashed lines) and the relative effects are presented in Table 2. Nine treatment comparisons yielded a significant association. The bulk of indirect evidence highlights the inferiority of monotherapies (CsA or MTX) over double or triple combination regimens (Table 2). Moreover, substitution of CsA for ATG was inferior to CsA/MTX (OR 12.4;95%CI 2.19–70, NNTH = 2) and addition of Pse to ATG/MTX was superior to ATG/MTX (OR 0.14; 95% CI 0.03–0.74, NNTB = 3). Finally combining sirolimus/tacrolimus with or without MTX was superior over CsA/MTX combination (OR 0.10; 95% CI 0.02–0.49, NNTB = 4). ATG add-on to CsA/MTX was comparable to tacrolimus/MTX (OR 1.02; 95% CI 0.49–2.12).

**Table 2:** Summary of indirect effects for the primary outcome (II-IV GvHD).

Prophylaxis	OR (95% CI)
Pse/CsA *vs*. MTX monotherapy	0.62 (0.21–1.85)
Tacrolimus/MTX *vs. MTX* monotherapy	**0.05 (0.01–0.27)**
Pse/CsA/MTX *vs.* MTX monotherapy	**0.10 (0.02–0.50)**
ATG/CsA/MTX *vs*. CsA monotherapy	**0.22 (0.11–0.45)**
Pse/ATG/MTX *vs*. CsA monotherapy	**0.19 (0.04–0.92)**
Tacrolimus/MTX *vs*. CsA monotherapy	**0.22 (0.11–0.41)**
MMF/CsA *vs*. CsA monotherapy	0.77 (0.20–2.93)
*ATG/MTX vs*. CsA/MTX	**12.4 (2.19–70)**
*Pse/CsA vs*. CsA/MTX	1.08 (0.44–2.64)
BDP/tacrolimus/MTX *vs.* CsA/MTX	0.42 (0.17–1.01)
Tacrolimus/MM*F vs.* CsA/MTX	0.43 (0.14–1.32)
*Tacrolimus/Sirolimus(±MTX) vs*. CsA/MTX	**0.10 (0.02–0.49)**
Pse/ATG/MTX *vs.* ATG/MTX	**0.14 (0.03–0.74)**
Pse/CsA/MTX *vs.* MTX monotherapy	**0.36 (0.15–0.87)**
Pse/CsA/MTX *vs.* ATG/CsA/MTX	1.62 (0.63–4.16)
ATG/CsA/MTX *vs*.Tacrolimus/MTX	1.02 (0.49–2.12)
MMF/CsA *vs*. Tacrolimus/MTX	3.55 (0.92–13.7)
BDP/Tacrolimus/MTX *vs.* Tacrolimus/MMF	0.96 (0.27–3.39)
Tacrolimus/Sirolimus/(±MTX) *vs*. Tacrolimus/MMF	0.22 (0.03–1.42)
BDP/Tacrolimus/MTX *vs. Tacrolimus/Sirolimus(±MTX)*	4.32 (0.77–24.17)
ATG/CsA/MTX *vs*. MMF/CsA	0.29 (0.07–1.15)

## Discussion

We performed a meta-analysis of randomized trials to quantify the effect of different preventive policies in acute GvHD prophylaxis after HSCT. Direct and indirect evidence further underscored the inferiority of CsA or MTX monotherapies over combined prophylaxis. Monotherapies represent outdated practices and are of no significance in clinical practice. However, their inclusion in this analysis is mandated for reason of completeness and integrity of the analysis. The CsA/MTX combination was the most widely adopted prophylaxis used and was considered the standard of care. There was substantial direct evidence derived from the myeloablative setting, that CsA/MTX outperforms CsA monotherapy. The evidence was deemed to be of high quality and supported the recommendation of CsA/MTX as the standard prophylaxis in myeloablative setting by the EBMT-ELN working group [5].

Interestingly, we found direct evidence on the superiority of tacrolimus/MTX over the standard CsA/MTX regimen. In the context of these findings, tacrolimus/MTX should also be another preferred strategy. However, tacrolimus is not widely used over CsA across Europe and clinical experience is limited [52]. For example, across the 72 centers that responded to a recent survey tacrolimus/MTX was ranked fourth (used merely in 5% of centers that responded to the survey), well below CsA/MTX (87%), CsA-MMF (11%) and CsA monotherapy (7%) [52]. The EBMT-ELN working group noted that the lack of experience in Europe precludes a firm recommendation; nevertheless tacrolimus/MTX is considered as equivalent alternative to CsA/MTX. A recent registry data analysis further supports the preferential use of tacrolimus/MTX over CsA/MTX-based regimens for siblings (adjusted OR 0.65;95%CI 0.53–0.80) or unrelated donors (adjusted OR 0.79;95% 0.67–0.94) [53]. It appears that European centers, most of which have extensive experience in using CsA-based prophylaxis, are reluctant to substitute CsA with tacrolimus. However, within the context of available evidence that change of policy may be warranted.

We also found high quality evidence that ATG add-on to CsA/MTX prophylaxis significantly reduced the risk of GvHD grade II-IV and may further justify the inclusion of ATG for unrelated donor transplantation [5]. A previous Cochrane review [54] has also demonstrated a significant decline in II-IV GvHD (risk ratio 0.68; 95% CI 0.55 to 0.85) after pooling all the pertinent studies, but no subgrouping was performed on the referent prophylaxis (MTX or CsA/MTX). As our data show, ATG has significant benefit as add-on to CsA/MTX, but no effect as add-on to MTX monotherapy. ATG add-on to CsA/MTX and tacrolimus/MTX (over the standard CsA/MTX) also demonstrated significant benefit regarding III-IV GvHD prevention. Moreover, indirect evidence suggested that ATG add-on to CsA/MTX is comparable to tacrolimus/MTX. Consequently, ATG/CsA/MTX should be regarded as equivalent to tacrolimus/MTX in terms of GvHD prevention. ATG use, as opposed to tacrolimus use, is popular across European centers [52] but was not widely adopted across American centers [55]. If ATG add-on is selected, caution on the policy of a center regarding CMV prevention is warranted, as ATG is an independent risk factor for CMV reactivation [56].

Of note is that sirolimus plus tacrolimus combination may outperform tacrolimus/MTX (direct evidence) and CsA/MTX (indirect evidence). However, the risk of post-transplant thrombotic microangiopathy (TMA) and sinusoidal obstruction syndrome [57], [58] are a concern and sirolimus dosage adjustment is strongly warranted [57]. The risk of sinusoidal obstruction syndrome further increases with busulfan-based conditioning regimens [58]. Nevertheless, sirolimus/tacrolimus combinations arise as potential alternatives to standard regimens, provided that the potential harms are taken into account.

Importantly, direct effects did not support the inclusion of add-on corticosteroids to the prophylaxis regimen, with overall effect pointing to insignificant decline. Corticosteroids are the mainstay of care for established acute GvHD, and early initiation is warranted at a dose of 2 mg/kg methylprednisolone upon clinical symptoms and signs (grade II or higher) by both randomized data [59] and the expert opinion [5]. However, it should be emphasized that the individual studies are clinically heterogeneous with respect to timing of corticosteroid administration, CsA dose and folinic acid rescue [31], [34], [48]. Moreover, corticosteroid add-on has resulted in late occurrence of acute GvHD [31], [48] and intensification of immunosuppression may lead to increased risk of relapse as shown in previous studies [60], [61]. The risks of corticosteroid treatment that include hypertension, infections or avascular bones necrosis may outweigh any potential benefits, and EBMT registry data have shown that such an aggressive approach may result in more deaths from infection and graft failures and reduced overall survival [62]. The lack of significant effects in preventing GvHD coupled with the risks of untoward effects and toxicities underscore that systemic corticosteroids should be not considered as part of the initial regimen for GvHD prophylaxis.

The effectiveness of MMF-based regimens remains an unresolved issue given that few randomized studies have compared MMF-based regimens in myeloablative allo-HSCT. The individual study data suggested the non-inferiority these MMF-based regimens but treatment comparators and donors differed across studies and data could not be pooled [23],[27]. We had no randomized trials on MMF-based prophylaxis in the RIC setting, but it should be noted that recent non-randomized data found no difference in II-IV aGVHD rates after RIC, between patients receiving MMF/CsA (38%) compared to CsA/MTX prophylaxis (33%, p = 0.5) [63]. The use of MMF/CsA is the most accepted prophylaxis in RIC across Europe, given the sustained complete chimerism and prolonged remission achieved after RIC [64].

Our findings are consistent with previous meta-analyses on the topic. More specifically, Ram et al. [65] documented the superiority of tacrolimus/MTX over CsA/MTX and the inferiority of CsA monotherapy over CsA/MTX. The authors found no effect from adding corticosteroids to prophylaxis. Also, Theurich et al. [54] noted a significant reduction of II-IV when ATG was added for GvHD prophylaxis, as we did. The consistency of our associations with previous studies strengthens the validity of our data. Moreover, our analysis provides some additional insights by finely stratifying the referent arms (this analysis permits relative effects to be derived for single agents and combinations) and provides relative effects for treatments that are not examined directly (indirect comparisons). In this context, our analysis is a comprehensive network of comparisons that provides –within the limits of the methodology- the best available evidence.

Our analysis did not address longitudinal outcomes, including relapse rates, non-relapse mortality and overall survival. Variability in the duration of follow-up, censored observations, lack of uniform reporting and between-study clinical heterogeneity (with regard to conditioning regimens, the use of TBI, BM/PBSCT transplantation and underlying conditions- all of which affect these outcomes [53], [66]–[69]), preclude a robust comparison of long-term effects.

A number of limitations should be noted. Clinical heterogeneity remains an important constraint. Meta-analysis cannot account for confounding variables and the reported relative effects are unadjusted for potentially influential covariates. Consequently, adjusting the effects for age, different conditioning regimens, the use of TBI, different dosing and way of administration of the same drug is not feasible. Additionally, the impact of differences in supportive therapy could not be addressed. Second, performance bias is a concern since, in most studies, arm assignment was not blinded to investigators, physicians or patients. Third, outcomes may alter depending on if GvHD was a primary or a secondary outcome in a contributing study. Fourth, the analysis refers to a single aspect of immunosuppressive regimen selection, that is the risk of GvHD. Other aspects that may influence the choice of immunosuppressive drugs such as age, donor type, degree of matching, and preparative regimens could not be accounted for. For example, T-cell depletion limits the problem of GvHD at the expense of relapse, whereas more effective regimens may have high mortality from infection negating the benefit of reduced GvHD. Fifth, III-IV GvHD was an under-reported outcome across studies, as 27% of trials did not report the pertinent data. The field of transplant medicine is moving towards individualizing treatment and this study adds to our knowledge, providing a framework of relative effects for regimens directly compared (direct effects) as well for those that were never compared in a randomized trial (indirect effects). This matrix of effects can serve as a basis for further research on the topic.

In summary, we systematically assessed the effects of GvHD prevention policies after transplantation. The direct and indirect evidence accumulated from randomized control trials ranked tacrolimus/MTX and ATG/CsA/MTX as outperformers of prevention policies, noted the potency of tacrolimus/sirolimus combination, verified the inferiority of monotherapies and highlighted the paucity of randomized data in specific settings, including non-myeloablative transplant recipients.

## Supporting Information

S1 Appendix **The appendix includes the following items: S1 Checklist.** PRISMA checklist. **S1** **Flow-Chart**. Flow-chart of study selection process. **S1 Table.** Characteristics of included studies. **S2 Table.** Quality assessment of the individual studies graded using the Cochrane Collaboration's tool, stratified by pairwise comparison: (a). MTX vs. CsA (b). MTX vs. CsA/MTX (c). Tacrolimus/MTX vs. CsA/MTX (d). CsA vs. CsA/MTX (e). Pse/Csa/MTX vs. CsA/MTX (f). Beclomethasone/tacrolimus/MTX vs. tacrolimus/MTX (g) Pse/CsA vs.CsA (h) ATG/CsA/MTX vs. CsA/MTX (i)ATG/MTX vs.MTX (j).Sirolimus/Tacrolimus(MTX) vs. Tacrolimus/MTX (k). MMF/Tacrolimus vs. Tacrolimus/MTX (l). MMF/CsA vs. CsA/MTX (m). Pse/CsA/MTX vs. Pse/CsA (n).Pse/ATG/MTX vs. MTX. **S3 Table.** GRADE Evidence Profile (EP) on the relative effects of pharmacologic prophylaxis on the risk of II-IV GvHD. **S4** **Table.** GRADE Summary of findings (SoF) table on the relative effects of pharmacologic prophylaxis on the risk of II-IV GvHD.
(DOCX)
