# Supplementary material for: Graft-Versus-Host Disease Prophylaxis after Transplantation: A Network Meta-Analysis
Source: PLoS One. 2014 Dec 8;9(12):e114735. doi: 10.1371/journal.pone.0114735 (PMC4259365; doi:10.1371/journal.pone.0114735)
Supplement: S1 Appendix — The appendix includes the following items: S1 Checklist. PRISMA checklist. S1 Flow-Chart. Flow-chart of study selection process. S1 Table. Characteristics of included studies. S2 Table. Quality assessment of the individual studies graded using the Cochrane Collaboration's tool, stratified by pairwise comparison: (a). MTX vs. CsA (b). MTX vs. CsA/MTX (c). Tacrolimus/MTX vs. CsA/MTX (d). CsA vs. CsA/MTX (e). Pse/Csa/MTX vs. CsA/MTX (f). Beclomethasone/tacrolimus/MTX vs. tacrolimus/MTX (g) Pse/CsA vs.CsA (h) ATG/CsA/MTX vs. CsA/MTX (i)ATG/MTX vs.MTX (j).Sirolimus/Tacrolimus(MTX) vs. Tacrolimus/MTX (k). MMF/Tacrolimus vs. Tacrolimus/MTX (l). MMF/CsA vs. CsA/MTX (m). Pse/CsA/MTX vs. Pse/CsA (n).Pse/ATG/MTX vs. MTX. S3 Table. GRADE Evidence Profile (EP) on the relative effects of pharmacologic prophylaxis on the risk of II-IV GvHD. S4 Table. GRADE Summary of findings (SoF) table on the relative effects of pharmacologic prophylaxis on the risk of II-IV GvHD. (DOCX) [file pone.0114735.s001.docx]

Checklist S1: PRISMA checklist

| **Section/topic** | **#** | **Checklist item** | **Reported on page #** |
| --- | --- | --- | --- |
| **TITLE** | | |  |
| Title | 1 | Identify the report as a systematic review, meta-analysis, or both. | p.1 |
| **ABSTRACT** | | |  |
| Structured summary | 2 | Provide a structured summary including, as applicable: background; objectives; data sources; study eligibility criteria, participants, and interventions; study appraisal and synthesis methods; results; limitations; conclusions and implications of key findings; systematic review registration number. | p.2 |
| **INTRODUCTION** | | |  |
| Rationale | 3 | Describe the rationale for the review in the context of what is already known. | p.4 |
| Objectives | 4 | Provide an explicit statement of questions being addressed with reference to participants, interventions, comparisons, outcomes, and study design (PICOS). | p.4 |
| **METHODS** | | |  |
| Protocol and registration | 5 | Indicate if a review protocol exists, if and where it can be accessed (e.g., Web address), and, if available, provide registration information including registration number. | Not applicable |
| Eligibility criteria | 6 | Specify study characteristics (e.g., PICOS, length of follow-up) and report characteristics (e.g., years considered, language, publication status) used as criteria for eligibility, giving rationale. | p.5, Methods |
| Information sources | 7 | Describe all information sources (e.g., databases with dates of coverage, contact with study authors to identify additional studies) in the search and date last searched. | p.5 Methods |
| Search | 8 | Present full electronic search strategy for at least one database, including any limits used, such that it could be repeated. | p.5, Methods |
| Study selection | 9 | State the process for selecting studies (i.e., screening, eligibility, included in systematic review, and, if applicable, included in the meta-analysis). | p.5,6, Methods |
| Data collection process | 10 | Describe method of data extraction from reports (e.g., piloted forms, independently, in duplicate) and any processes for obtaining and confirming data from investigators. | p.5,6, Methods |
| Data items | 11 | List and define all variables for which data were sought (e.g., PICOS, funding sources) and any assumptions and simplifications made. | p.5, Methods |
| Risk of bias in individual studies | 12 | Describe methods used for assessing risk of bias of individual studies (including specification of whether this was done at the study or outcome level), and how this information is to be used in any data synthesis. | p.6,7, Methods |
| Summary measures | 13 | State the principal summary measures (e.g., risk ratio, difference in means). | p.6,7, Methods |
| Synthesis of results | 14 | Describe the methods of handling data and combining results of studies, if done, including measures of consistency (e.g., I^2^) for each meta-analysis. | p.6,7, Methods |

| Risk of bias across studies | 15 | Specify any assessment of risk of bias that may affect the cumulative evidence (e.g., publication bias, selective reporting within studies). | p.6, Table S4 |
| --- | --- | --- | --- |
| Additional analyses | 16 | Describe methods of additional analyses (e.g., sensitivity or subgroup analyses, meta-regression), if done, indicating which were pre-specified. | p. 6,7, Methods |
| **RESULTS** | | |  |
| Study selection | 17 | Give numbers of studies screened, assessed for eligibility, and included in the review, with reasons for exclusions at each stage, ideally with a flow diagram. | p.7, Results |
| Study characteristics | 18 | For each study, present characteristics for which data were extracted (e.g., study size, PICOS, follow-up period) and provide the citations. | p.7,8,Results |
| Risk of bias within studies | 19 | Present data on risk of bias of each study and, if available, any outcome level assessment (see item 12). | p. 8, Table S4 |
| Results of individual studies | 20 | For all outcomes considered (benefits or harms), present, for each study: (a) simple summary data for each intervention group (b) effect estimates and confidence intervals, ideally with a forest plot. | Figure 2&3, Table 1 |
| Synthesis of results | 21 | Present results of each meta-analysis done, including confidence intervals and measures of consistency. | Figure 2&3, Table 2, p. 9-11 |
| Risk of bias across studies | 22 | Present results of any assessment of risk of bias across studies (see Item 15). | Table S4, S5, S6 |
| Additional analysis | 23 | Give results of additional analyses, if done (e.g., sensitivity or subgroup analyses, meta-regression [see Item 16]). | p. 9-12 |
| **DISCUSSION** | | |  |
| Summary of evidence | 24 | Summarize the main findings including the strength of evidence for each main outcome; consider their relevance to key groups (e.g., healthcare providers, users, and policy makers). | p. 12-15, Discussion |
| Limitations | 25 | Discuss limitations at study and outcome level (e.g., risk of bias), and at review-level (e.g., incomplete retrieval of identified research, reporting bias). | p. 15-16,  Discussion |
| Conclusions | 26 | Provide a general interpretation of the results in the context of other evidence, and implications for future research. | p. 15,16-17, Discussion |
| **FUNDING** | | |  |
| Funding | 27 | Describe sources of funding for the systematic review and other support (e.g., supply of data); role of funders for the systematic review. | None |

Flow-chart S1: Flow-chart of study selection process

Articles included

In the analysis (n=32)

Studies added (n=0) from:

- Manual search of reference lists

Full-text articles excluded,

with reasons
(n =21 )

-n=12 had extension.overlapping data

-n=4 randomzed drugs not advocated by EBMT-ELN guidelines

-n=4 evaluated different dose/scheme of the same drug

-n=1 not truly randomized (matched-case analysis)

Records screened

(title & abstract)
(n =1028 )

Articles eligible for analysis (n=32)

Full-text articles assessed for eligibility
(n = 53)

Records excluded
(n = 975)

Records after duplicates removed
(n =1028)

Records identified through

The Cochrane Library search
(n =694)

Records identified through

PubMed search
(n =769)

Table S1: Characteristics of included studies

| **Author** | **Year** | **Population** | **Donor** | **Setting** | **Source** | **Underlying condition** | | **Arm 1** | **Arm 2** | |  |
| --- | --- | --- | --- | --- | --- | --- | --- | --- | --- | --- | --- |
| Pulsipher [^20^](#_ENREF_20) | 2014 | Children (1-21) | SB (54.5%);  UD (42.7%) | TBI, Thiotepa and CY | BM  PBSC  chord | ALL (100%) | | MTX 5 mg/m^2^ IV on days +1, +3, +6 for all stem cell sources and day +11 for unrelated BM or PBSC  and TAC loading dose on day -2 target levels 5-12ng/ml **and SIR day +0 at a dose of 4 mg/m2, target levels 3-12ng/ml** | MTX 5 mg/m^2^ IV on days +1, +3, +6 for all stem cell sources and day +11 for unrelated BM or PBSC  and TAC loading dose on day -2 target levels 5-12ng/ml | |  |
| Martin[^22^](#_ENREF_22) | 2012 | All age groups | SB (36.2%)  (34.8%); UD (53.8%) | TBI and CY; CY and Bu | BM (25.4%); PBSC (74.6%) | AML (41.3%); ALL (22.5%); CML (9.4%); MDS or MPS (24.6%); Other (2.2%) | | MTX 15mg/m^2^ i.v. on day +1 and 10mg/m^2^ on days +3, +6, +11 and TAC with starting dose 0.03mg/kg/d i.v. on day -1 and target trough level 10-40ng/ml **and BDP as 1mg immediate-release formulation plus 1mg delayed release p.os. 4xd beginning at the start of MCR through day +75** | MTX 15mg/m^2^ i.v. on day +1 and 10mg/m^2^ on days +3, +6, +11 and TAC with starting dose 0.03mg/kg/d i.v. on day -1 and target trough level 10-40ng/ml | |  |
| Pidala [^21^](#_ENREF_21) | 2012 | 23-69 | SB (47.3%);  UD (52.7%) | Bu and Flu | PBSC (100%) | AML (31.1%);  ALL (20.3%);  CLL (9.5%);  MDS (12.2%)  MM (10.8%)  NHL (6.8%) | | TAC 0.02mg/kg/day from day -3 until hospital discharge with conversion to p.os **and SIR 9mg p.os loading dose on day -1, following maintenance with target 5-14ng/ml, continued for at least a year after HSCT** | TAC 0.02mg/kg/day from day -3 until hospital discharge with conversion to p.os **and**  **MTX 15mg/m^2^ iv on day +1, 10mg/m^2^ iv on day +3, +6, +11** | | |
| Perkins[^23^](#_ENREF_23) | 2010 | Adults (23-70) | SB (48.3%)  UD (51.7%) | Flu and Bu | PBSC (100%) | AML (37.1%)  MDS (25.8%)  ALL (9.0%)  Other (28.1%) | | TAC 0.03 mg/kg/day iv infusion from day -3, When the patient was able to tolerate oral medications, p.os dosing 2xd, with target trough levels 5-15ng/ml **and**  **MMF 30 mg/kg/d i.v. in 2 divided doses from day +0 at least 2 hours after the end of the infusion of donor cells. Po formulation when patients were able to tolerate oral medications.** | TAC 0.03 mg/kg/day iv infusion from day -3, When the patient was able to tolerate oral medications, p.os dosing 2xd, with target trough levels 5-15ng/ml **and MTX 15mg/m^2^ iv on day +1, 10mg/m^2^ iv on day +3, +6, +11** | | |
| Bacigalupo[^25^](#_ENREF_25) | 2010 | All age groups | UD (85.3%); SB (24.7%) | CY and TBI (58.0%); RIC (42.0%) | BM (79.0%); PBSC (21.0%) | AL (49.0%); CML (15.0%); Other (36.0%) | | CsA 1 mg/kg i.v. from day -7, and  MTX 15mg/m^2^ on day +1 and 10mg/m^2^ on days +3, +6  and +11 and **ATG 1.25 mg/kg i.v. on days +7, +9** | CsA 1 mg/kg i.v. from day -7, and  MTX 15 mg/m^2^ on day +1 and 10 mg/m^2^ on days +3, +6  and +11 | |  |
| Finke[^24^](#_ENREF_24) | 2009 | 18-60 | UD (100.0%) | TBI and CY(50.7%); Bu and CY (25.9%); TBI, VP-16 and CY (8.5%); Other (14.9%) | BM (18.4%); PBSC (81.6%) | AML (34.8%); ALL (50.2%); CML (8.5%); MDS (5.0%); MF (1.5%) | | CsA i.v. from day -1 (target trough concentrations ≥200ng/ml) and MTX 15mg/m^2^ on day +1 and 10mg/m^2^ on days +3, +6 and +11 and **ATG 20 mg/kg i.v. on days -1, -2 and -3** | CsA i.v. from day -1 (target trough concentrations ≥200ng/ml) and MTX 15mg/m^2^ on day +1 and 10mg/m^2^ on days +3, +6 and +11 | |  |
| Champlin[^26^](#_ENREF_26) | 2007 | ≤60 | SB (100%) | CY | BM (96.9%); PBSC (1.5%); BM and PBSC (0.8%) | AA (100%) | | CsA and MTX and **ATG 30 mg/kg i.v. on days −5 to −3** | CsA and MTX | |  |
| Lee[^28^](#_ENREF_28) | 2004 | 15-50 | SB (100.0%) | CY and Bu | BM (100.0%) | AL (66.3%); MDS (6.2%); CML (27.5%) | | CsA 1.5mg/kg i.v. every 12h from day -1 and were switched to oral when oral intake was feasible **and MTX 15mg/m^2^ i.v. on day -1 and 10mg/m^2^ on days +3, +6 and +11 with target trough level 100-300ng/ml** | CsA 1.5mg/kg i.v. every 12h from day -1 and were switched to oral when oral intake was feasible | |  |
| Bolwell[^27^](#_ENREF_27) | 2004 | Adult (16-62) | SB (100%) | Bu and CY | BM (100.0%) | AML (72.5%)  NHL (12.5%)  Other (15%) | | CsA 300mg/m^2^ by continuous iv infusion daily from day -1 until hematopoietic engraftment, and thereafter p.os attempting to maintain a therapeutic trough level of 200 **and MMF 500mg 3 times daily from day +1 through day +100** | CsA 300mg/m^2^ by continuous iv infusion daily from day -1 until hematopoietic engraftment, and thereafter p.os attempting to maintain a therapeutic trough level of 200 **and MTX 5mg/m^2^ iv on day +1, +3, +6, and +11** | | |
| Bacigalupo[^29^](#_ENREF_29) | 2001 | All age groups | UD (100%) | CY and TBI | BM (100.0%) | Hematologic malignancies (100.0%) | | CsA 2mg/kg/d i.v. on from day -1 until +20, then 6 to 10mg/kg/d p.os for at least 1y and MTX 15mg/m^2^ i.v. on days +1 and 10mg/m^2^ on days +3, +6 and +11and **1 mg/kg i.v. P before the infusion of ATG and 3.75 mg/kg ATG i.v.**  **on**  **days -4 and -3** | CsA 2mg/kg/d i.v. on from day -1 until +20, then 6 to 10mg/kg/d p.os for at least 1y and MTX 15mg/m^2^ i.v. on days +1 and 10mg/m^2^ on days +3, +6 and +11 | |  |
| Bacigalupo[^29^](#_ENREF_29) | 2001 | All age groups | UD (100%) | CY and TBI | BM (100.0%) | Hematologic malignancies (100.0%) | | CsA 2mg/kg/d i.v. on from day -1 until +20, then 6 to 10mg/kg/d p.os for at least 1y and MTX 15mg/m^2^ i.v. on days +1 and 10mg/m^2^ on days +3, +6 and +11 and **1 mg/kg i.v. P before the infusion of ATG and 3.75 mg/kg ATG i.v.**  **on**  **days -5, -4, -3, -2** | CsA 2mg/kg/d i.v. on from day -1 until +20, then 6 to 10mg/kg/d p.os for at least 1y and MTX 15mg/m^2^ i.v.on days +1 and 10mg/m^2^ on days +3, +6 and +11 | |  |
| Hiraoka[^30^](#_ENREF_30) | 2001 | All age groups | SB (94.6%) | Varied | BM (100.0%) | Leukemia (76.3%); AA (4.6%); MDS (10.7%); Other (8.4%) | | **TAC with starting dose 0.075mg/kg p.os 2xd on day -1 with target trough level 20-25ng/ml for a 2 to 3 week period after transplantation and 10-15ng/ml thereafter** | **CsA with starting dose 0.05mg/kg/d i.v. on day -1 with target trough level 20-25ng/ml for a 2 to 3 week period after transplantation and 10-15ng/ml thereafter** | |  |
| Ruutu[^31^](#_ENREF_31) | 2000 | ≥18 | SB (100.0%) | CY and TBI; CY and Bu | BM (100.0%) | AML (39.8%); CML (25.9%); ALL (13.9%); MDS (8.3%); MM (7.4%); NHL (2.8%); CLL (1.9%) | | CsA 3mg/kg/d i.v. beginning on day -1 and MTX 15mg/m^2^ i.v. on day +1 and 10mg/m^2^ on days +3, +6 and +11 **and MP 0.5 mg/kg p.os. on days +14 to +20, 1mg/kg on days +21 to +34 and 0.5 mg/kg on days +35 to +48 with dose tapered thereafter and discontinued on day 110** | CsA 3mg/kg/d i.v. beginning on day -1 and MTX 15mg/m^2^ i.v. on day +1 and 10mg/m^2^ on days +3, +6 and +11 | |  |
| Chao[^34^](#_ENREF_34) | 2000 | All age groups | SB (100.0%) | TBI and VP-16 (93.0%); TBI and CY (7.0%) | BM (100.0%) | CML (46.8%); AML (31.7%); ALL (21.5%) | | CsA 5mg/kg i.v. on days -2 to +3, 3mg/kg on days +4 to +14 and 3.75mg/kg on days +15 to +35 and p.os thereafter until day +180 and MTX 15mg/m^2^ i.v. on day +1 and 10mg/m^2^ on days +3, +6 and +11 and **MP 0.25mg/kg i.v. on days +7 to +14 and 0.5mg/kg on days +15 to +28 with P p.os thereafter on tapered dose and discontinued on day +180** | CsA 5mg/kg i.v. on days -2 to +3, 3mg/kg on days +4 to +14 and 3.75mg/kg on days +15 to +35 and p.os thereafter until day +180, and MTX 15mg/m^2^ i.v. on day +1 and 10mg/m^2^ on days +3, +6 and +11 | |  |
| Nash[^32^](#_ENREF_32) | 2000 | All age groups | UD (100.0%) | TBI or Bu | BM (100.0%) | CML (49.4%); ALL (11.1%); AML (21.7%); NHL (1.7%); AA (3.3%); MDS (12.2%); Other (0.6%) | | MTX 15mg/m^2^ i.v. on day +1 and 10mg/m^2^ on days +3, +6 and +11, **TAC with starting dose 0.03mg/kg i.v. on day -1 with target trough level 10-30ng/ml** | MTX 15mg/m^2^ i.v. on day +1 and 10mg/m^2^ on days +3, +6 and +11, **CsA with starting dose 3mg/kg i.v. on day -1 with target trough level 150-450ng/ml** | |  |
| Locatelli[^33^](#_ENREF_33) | 2000 | 2-50 | SB (100.0%) | CY | BM (100.0%) | AA (100.0%) | | CsA 3mg/kg/d i.v. from day -1 and were switched to oral when oral intake was feasible **and MTX from day -1 with a dose of 8mg/m^2^ on days +1, +3, +6 and +11** | CsA 3mg/kg/d i.v. from day -1 and were switched to oral when oral intake was feasible | |  |
| Ratanatharathorn[^35^](#_ENREF_35) | 1998 | ≥12 | SB (100.0%) | Varied | BM (100.0%) | MDS (6.1%); ALL (9.7%); AML (27.1%); CML (32.8%); NHL (8.8%); MM (7.0%);HL (1.2%); CLL (3.0%) Other (4.3%) | | MTX 15mg/m^2^ i.v. on day +1 and 10mg/m^2^ on days +3, +6 and +11 **and TAC with starting dose 0.03mg/kg/d i.v. on day -1 with target trough level 10-40ng/ml** | MTX 15mg/m^2^ i.v. on day +1 and 10mg/m^2^ on days +3, +6 and +**11 and CsA with starting dose 3mg/kg/d i.v. on day -1 with target trough level 150-450 ng/ml** | |  |
| Zikos[^36^](#_ENREF_36) | 1998 | 16-50 | SB (100.0%) | CY and TBI (100.0%) | BM (100.0%) | AML (100.0%) | | CsA 1mg/kg i.v. from day -1 to day +20 **and MTX 10mg/m^2^ i.v. on day +1 and 8mg/m^2^ on days +3, +6, +11** | CsA 1mg/kg i.v. from day -1 to day +20 | |  |
| Deeg[^37^](#_ENREF_37) | 1997 | All age groups | SB (100.0%) | CY and TBI (53.3%); Bu, CY and TBI (27.9%); Bu and CY (8.2%); CY and TBI (1.6%); Other (9.0%) | BM (100.0%) | ALL (23.0%); ANL (14.8%); CML (3.3%); MDS (21.3%); Lym (33.6%); Other (4.1%) | | CsA 5mg/kg/d i.v. on days -1 through +3 and 3mg/kg/d on days +4 through +14, 3.75mg/kg/d on days +15 through +35 and continued p.os. in tapered doses after discharge through day +180 and **MP 0.25mg/kg 2xd i.v. on days +7 through +14, 0.5mg/kg 2xd on days +15 through  +28 and tapered thereafte**r | CsA 5mg/kg/d i.v. on days -1 through +3 and 3mg/kg/d on days +4 through +14, 3.75mg/kg/d on days +15 through +35 and continued p.os. in tapered doses after discharge through day +180 | |  |
| Chao [^38^](#_ENREF_38) | 1993 | All age groups | SB (100.0%) | TBI and VP-16 | BM (100%) | AML (33.6%);  ALL (18.1%);  CML (45.0%);  Other (3.4%) | | CsA 5mg/kg iv from day -2, 3mg/kg from day +4 through+15, 3.75mg/ kg from day +15 through +36, CsA 10mg/kg p.os from day +36 and slowly tapering through day +180 and MP 0.5mg/kg iv from day+7, 1mg/kg iv from day +15and P 0.8mg/kg p.os. from day +29 and tapering through day +180 **and MTX 15mg/m^2^ iv on day +1, 10mg/m^2^ iv on day +3, +6 and +11** | CsA 5mg/kg iv from day -2, 3mg/kg from day +4 through+15, 3.75mg/ kg from day +15 through +36, CsA 10mg/kg p.os from day +36 and slowly tapering through day +180 and MP 0.5mg/kg iv from day+7, 1mg/kg iv from day +15 and P 0.8mg/kg p.os. from day +29 and tapering through day +180 | | |
| Atkinson [^49^](#_ENREF_49) | 1991 | ≥15 | SB (100.0%) | CY and Bu; CY and TBI | BM (100.0%) | ANL (39.0%); ALL (14.6%); CML (43.9%); Lym (2.5%) | | CsA 3mg/kg/d i.v. on days -1 to +5 and then switched to p.os and MTX 7.5mg/m^2^ on days +1, +3, +6 and +11 and **MP 0.5mg/kg i.v. 2x daily on alternate days starting on day 0 through day +20 and thereafter P at the same dose p.os until day +30** | CsA 3mg/kg/d i.v. on days -1 to +5 and then switched to p.os and MTX 7.5mg/m^2^ on days +1, +3, +6 and +11 | |  |
| Storb[^48^](#_ENREF_48) | 1990 | All age groups | SB (100.0%) | CY and TBI; CY | BM (100.0%) | ANL (42.9%); CML (38.8%); MDS (9.5%); AA (8.8%) | | MTX 15mg/m2 i.v. on day +1 and 10mg/m2 on days +3, +6 and +11 and CsA 1.5mg/kg i.v. on day -1 and every 12 h until recovering from conditioning regimen GI toxicity and thereafter 6.25mg/kg p.os every 12h until day +60 **and MP 1mg/kg i.v. on days 0 through +22 and 0.5mg/kg i.v. on days +23 to +35 and discontinued thereafter** | MTX 15mg/m2 i.v. on day +1 and 10mg/m2 on days +3, +6 and +11 and CsA 1.5mg/kg i.v. on day -1 and every 12 h until recovering from conditioning regimen GI toxicity and thereafter 6.25mg/kh p.os every 12h until day +60 | |  |
| Mrsić[^47^](#_ENREF_47) | 1990 | All age groups (5-45) | SB (100%) | CY and TBI | BM (100%) | AML (39.5%)  ALL (31.6%)  CML (28.9%) | CsA 10mg/kg i.v. infusion on day -1, 5mg/kg iv infusion on day 0, +1, 3mg iv infusion **and**  **MTX 15mg/m^2^ iv on day +1, 10mg/m^2^ iv on day +3, +6, +11** | | CsA 10mg/kg i.v. infusion on day -1, 5mg/kg iv infusion on day 0, +1, 3mg iv infusion. |  |  |
| Torres[^46^](#_ENREF_46) | 1989 | Adults | SB (100%) | CY and TBI  And daunorubicin for CML | BM (100%) | ANL (29.8%);  ALL (49.1%);  CML (15.8%);  AA (5.3%) | **CsA 2mg/kg iv bid, after GI recovery 6.25mg/kg 2xd p.os. after day +70 taper and stop on day +100, except for AA patients stopped on day +180** | | **MTX 15mg/m^2^ iv on day +1, 10mg/m^2^ iv on day +3, +6, +11 and weekly until day +100** |  |  |
| Ringdén[^44^](#_ENREF_44) | 1986 | All age groups | SB (100%) | CY,  MTX and TBI | BM (100%) | AL (69.5%);  CML (23.7%);  Other (6.8%) | | **MTX 15mg/m^2^ iv on day +1, 10mg/m^2^ iv on day +3, +6, +11, +18, +25 and every 2 weeks until day +95**. | **CsA 5mg/kg/d iv on day -1, then when able to tolerate p.os treatment 12.5mg/kg/day for 6months, and then decreased by 2mg/kg/d every alternate month** | | |
| Biggs[^42^](#_ENREF_42) | 1986 | All age groups | SB (100%) | CY and TBI | BM (100%) | ANL (75%), ALL (25%) | | **MTX 15mg/m^2^ i.v. on day +1, 10mg/m^2^ iv on day +3, +6, +11 and weekly until day +100** | **CsA 25mg/kg/d from day -1 to +4, then 12.5 mg/kg/d from +5 to +49, then reduced 5% per week until day +180** | | |
| Storb [^43^](#_ENREF_43) | 1986 | All age groups | SB (100%) | CY and TBI | BM (100%) | ANL, CML | | CsA on the day -1, 1.5 mg/kg i.v. 2xd until recovery from GI toxicity, then p.os 6.25 mg/kg 2xd until day +50 **and MTX 15mg/m^2^ iv on day +1, 10mg/m^2^ iv on day +3, +6, +11** | CsA on the day -1, 1.5 mg/kg i.v. 2xd until recovery from GI toxicity, then p.os 6.25 mg/kg 2xd until day +50 | | |
| Storb[^41^](#_ENREF_41) | 1986 | All age groups | SB (100%) | CY | BM (100%) | AA (100%) | | MTX 15mg/m^2^ i.v. on day +1, 10mg/m^2^ iv on day +3, +6, +11  **and CsA 1.5mg/kg every 12 h from day -1 until recovery, 6.25 mg/kg every 12 hours until day +50, then tapering for 6months** | MTX 15mg/m^2^ i.v. on day +1, 10mg/m^2^ iv on day +3, +6, +11, +18 and once weekly till day +102 | | |
| Deeg [^40^](#_ENREF_40) | 1985 | All age groups | SB (100%) | CY, MTX and TBI | BM (100%) | ALL | | **CsA 1.5 mg/kg iv bid until recovery from GI toxicity and then p.os 6.25 mg/kg bid until day +50** | **MTX 15mg/m^2^ iv on day +1, 10mg/m^2^ iv on day +3, +6, +11, +18, +25 and every week up to day +95** | | |
| Storb [^45^](#_ENREF_45) | 1985 | All age groups | SB (100%) | CY and TBI | BM (100%) | CML | | **CsA started on day -1 either p.os 6.25 mg/kg 2xd or i.v. 1.5mg/kg 2xd, full dose until day 50 afterwards 5% decrease per week until 6 months post transplantation**. | **MTX 15mg/m^2^ iv on day +1, 10mg/m^2^ iv on day +3, +6, +11, +18, +25, +39, +53, +67, +81, +95** | | |
| Ramsey [^39^](#_ENREF_39) | 1982 | All age groups | SB (100.0%) | MCR | BM (100%) | AA (40.3%); ALL (25.4%); ANL (16.4%); Lym (10.4%); CML (7.5%) | | MTX 15mg/m^2^ i.v. on day +1 and 10mg/m^2^ on days +3, +6 and +11 and weekly until day +100  **and ATG 15 mg/kg i.v. on days +8, +10, +12, +14, +16, +18, +20 and pse 40mg/m^2^ p.os on days 8 through 20** | MTX 15mg/m^2^ i.v. on day +1 and 10mg/m^2^ on days +3, +6 and +11 and weekly until day +100 | | |
| Doney [^50^](#_ENREF_50) | 1980 | All age groups | SB (100.0%) | CY and TBI; CY, TBI and Bu; Other | BM (100.0%) | Hematologic malignancies (100.0%) | | MTX 15mg/m^2^ i.v. on day+1 and 10mg/m^2^ on days +3, +6 and +11 and weekly until day +102 **and ATG 10mg/kg i.v. every other day for 6 doses beginning from day +7** | MTX 15mg/m^2^ i.v. on day+1 and 10mg/m^2^ on days +3, +6 and +11 and weekly until day +102 | |  |
| Weiden[^51^](#_ENREF_51) | 1979 | All age groups | SB (100.0%) | CY and TBI; CY, TBI and dimethyl myrelan; Other | BM (100.0%) | AML  (41.1%); ALL (42.9%); CML (8.9%); LLL (7.1%) | | MTX 15mg/m2 i.v. on day +1 and 10mg/m2 on days +3, +6 and +11 and then weekly until day +102 and **ATG 7mg/kg i.v. every other day for 6 doses beginning after marrow engraftment** | MTX 15mg/m2 i.v. on day +1 and 10mg/m2 on days +3, +6 and +11 and then weekly until day +102 | |  |

Annotation: CY=cyclophosphamide; TBI=total body irradiation; Bu=busulfan; VP-16=etoposide; BM=bone marrow; PBSC=peripheral blood stem-cells; TAC=tacrolimus; MTX=methotrexate; ATG=antithymocyte globulin; MP=methylprednisolone; CsA=cyclosporine A

Table S2: Quality assessment of the individual studies graded using the Cochrane Collaboration’s tool, stratified by pairwise comparison.


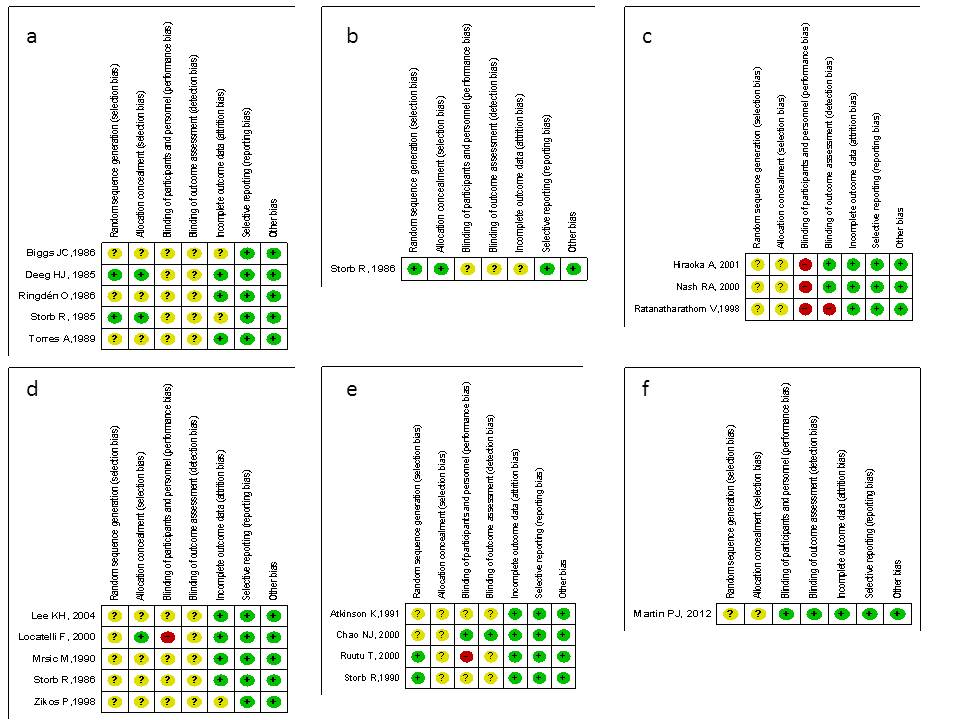


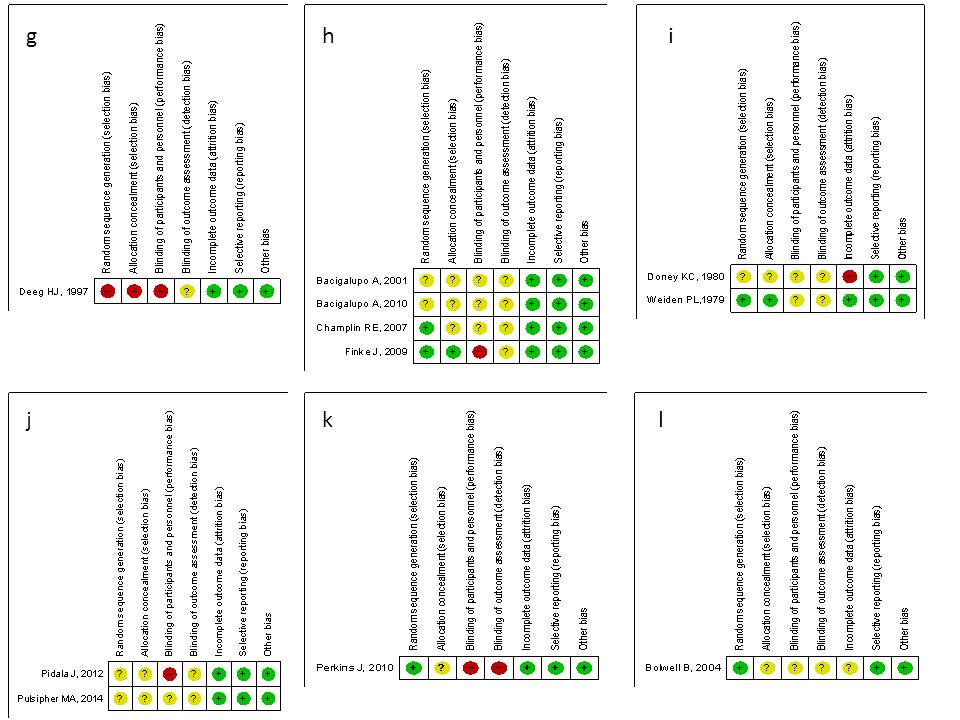


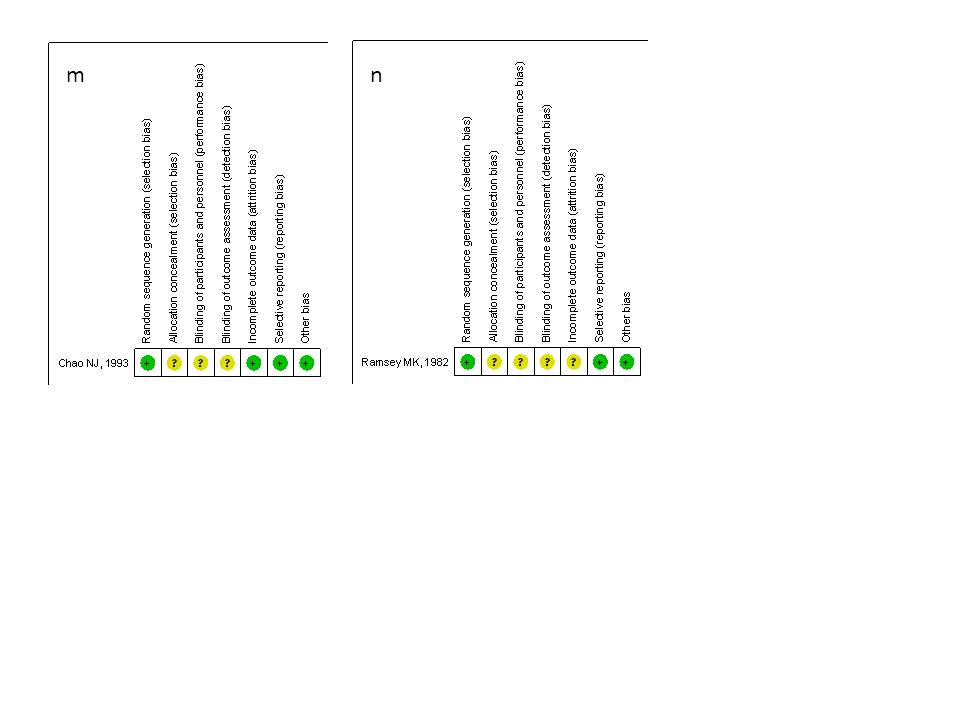


**Annotation:**

(**a**). MTX *vs.* CsA (**b**). MTX *vs*. CsA/MTX (**c**). Tacrolimus/MTX *vs*. CsA/MTX (**d**). CsA *vs*. CsA/MTX (**e**). Pse/Csa/MTX *vs*. CsA/MTX (**f**). Beclomethasone/tacrolimus/MTX *vs*. tacrolimus/MTX (**g**) Pse/CsA *vs.*CsA (**h**) ATG/CsA/MTX *vs.* CsA/MTX (**i**)ATG/MTX *vs.*MTX (**j**).Sirolimus/Tacrolimus(MTX) vs. Tacrolimus/MTX (**k**). MMF/Tacrolimus vs. Tacrolimus/MTX (**l**). MMF/CsA vs. CsA/MTX (**m**). Pse/CsA/MTX vs. Pse/CsA (**n**).Pse/ATG/MTX vs. MTX

Pse= systemic corticosteroids (including methylprednisolone, prednisone, and /or prednisolone)

Tables S3,4: GRADE rating of quality of evidence

Table S3: GRADE Evidence Profile (EP) on the relative effects of pharmacologic prophylaxis on the risk of II-IV GvHD

| **Quality assessment** |  |  |  |  |  |
| --- | --- | --- | --- | --- | --- |
| **Pairwise comparison** | **Limitations** | **Inconsistency** | **Indirectness** | **Imprecision** | **Publication bias** |
| *MTX vs. CsA* |  |  |  |  |  |
| 5 Randomized Trials | No serious limitations† | Serious  inconsistency | No serious  indirectness | Serious  Imprecision | Not detected |
| *MTX vs. CsA/MTX* |  |  |  |  |  |
| 1 Randomized Trial | No serious limitations† | No serious  inconsistency | No serious  indirectness | Serious  Imprecision | Not detected |
| *Tacrolimus/MTX vs. CsA/MTX* |  |  |  |  |  |
| 3 Randomized Trials | No serious limitations† | No serious  inconsistency | No serious  indirectness | No  imprecision | Not detected |
| *CsA vs. CsA/MTX* |  |  |  |  |  |
| 5 Randomized Trials | No serious limitations† | No serious  inconsistency | No serious  indirectness | Serious  Imprecision | Not detected |
| *Corticosteroid add-on*  *(Pse/CsA/MTX vs, CsA/MTX)* |  |  |  |  |  |
| 4 Randomized Trials | No serious limitations† | Serious  inconsistency | No serious  indirectness | No  Imprecision | Not detected |
| *Corticosteroid add-on*  *(BDP/Tacrolimus/MTX vs. Tacrolimus/MTX)* |  |  |  |  |  |
| 1 Randomized Trial | No serious limitations† | No serious  inconsistency | No serious  indirectness | Serious  Imprecision | Not detected |
| *Corticosteroid add-on (Pse/CsA vs. CsA)* |  |  |  |  |  |
| 1 Randomized Trial | Serious limitations†† | No serious  inconsistency | No serious  indirectness | Serious  Imprecision | Not detected |
| *ATG add-on (ATG/CsA/MTX vs. CsA/MTX)* |  |  |  |  |  |
| 4 Randomized trials | No serious limitations† | No serious  inconsistency | No serious  indirectness | No  Imprecision | Not detected |
| *ATG add-on (ATG/MTX vs. MTX)* |  |  |  |  |  |
| 2 Randomized Trials | No serious limitations† | No serious  inconsistency | No serious  indirectness | Serious  Imprecision | Not detected |
| *Sirolimus-based vs. Tacrolimus/MTX* |  |  |  |  |  |
| 2 Randomized Trials | No serious limitations† | Serious  inconsistency | No serious  indirectness | Serious  Imprecision | Not detected |
| *MMF/Tacrolimus vs. Tacrolimus/MTX* |  |  |  |  |  |
| 1 Randomized Trial | No serious limitations† | No serious  inconsistency | No serious  indirectness | Serious  Imprecision | Not detected |
| *MMF/CsA vs. CsA/MTX* |  |  |  |  |  |
| 1 Randomized Trial | No serious limitations† | No serious  inconsistency | No serious  indirectness | Serious  Imprecision | Not detected |
| *Pse/CsA/MTX vs. Pse/CsA* |  |  |  |  |  |
| 1 Randomized Trial | No serious limitations† | No serious  inconsistency | No serious  indirectness | Serious  Imprecision | Not detected |
| *Pse/ATG/MTX vs. MTX* |  |  |  |  |  |
| 1 Randomized Trial | No serious limitations† | No serious  inconsistency | No serious  indirectness | Serious  Imprecision | Not detected |

†high quality of the majority of individual studies (most items with low or unclear risk of bias across studies). The risk of bias was deemed low to unclear, and quality rating was not downgraded. †† downgraded one category (most items with high or unclear risk of bias)

Pse= systemic corticosteroids (including methylprednisolone, prednisone, and /or prednisolone); BDP=beclomethasone propionate

.

Table S4: GRADE Summary of findings (SoF) table on the relative effects of pharmacologic prophylaxis on the risk of II-IV GvHD.

| **Prophylaxis** | **relative effect (OR; 95% CI)** | **Number of participants (studies)** | **Quality of evidence (GRADE)** | **Comment** |
| --- | --- | --- | --- | --- |
| *Outcome: II-IV GvHD* |  |  |  |  |
| MTX vs. CsA | 0.85 (0.40-1.82) | 273 (5) | Low | ^2,3^ |
| MTX *vs. CsA/MTX* | 7.8 (1.79-34.07) | 44 (1) | High | ^1,3^ |
| Tacrolimus/MTX vs. CsA/MTX | 0.44 (0.27-0.70) | 640 (3) | High |  |
| CsA vs. CsA/MTX | 2.03 (1.31-3.15) | 378 (5) | Moderate | ^3^ |
| *Corticosteroid add-on* |  |  |  |  |
| Pse/CsA/MTX *vs,* CsA/MTX | 0.73 (0.34-1.57) | 482 (4) | Moderate | ^2^ |
| BDP/Tacrolimus/MTX *vs.* Tacrolimus/MTX | 0.95 (0.45-2.02) | 138 (1) | Moderate | ^3^ |
| Pse/CsA *vs. CsA* | 0.53 (0.24-1.15) | 120 (1) | Low | ^3,4^ |
| *ATG add-on* |  |  |  |  |
| ATG/CsA/MTX *vs*. CsA/MTX | 0.45 (0.26-0.78) | 440 (4) | High |  |
| ATG/MTX *vs. MTX* | 1.61 (0.64-4.07) | 128 (2) | Moderate | ^3^ |
| Sirolimus/Tacrolimus(MTX) *vs*. Tacrolimus/MTX | 0.22 (0.05-1.11) | 217 (2) | Moderate | ^1,2,3^ |
| MMF/Tacrolimus vs. Tacrolimus/MTX | 0.99 (0.36-2.74) | 40 (1) | Moderate | ^3^ |
| MMF/CsA *vs.* CsA/MTX | 1.56 (0.44-5.53) | 89 (1) | Moderate | ^3^ |
| Pse/CsA/MTX *vs.* Pse/CsA | 0.35 (0.13-0.89) | 149 (1) | High | ^1,3^ |
| Pse/ATG/MTX *vs.* MTX | 0.23 (0.06-0.90) | 67 (1) | High | ^1,3^ |

^1^ Upgraded one category due to large effect

^2^ Downgraded by one category due to inconsistency

^3^Downgraded by one category, optimal information size (OIS) was not reached (for α=0.05, power=0.80, effect difference 30%)

^4^Downgraded due to serious limitations

Pse= systemic corticosteroids (including methylprednisolone, prednisone, and /or prednisolone); BDP=beclomethasone propionate

.
